# Supplementary material for: DMRforPairs: identifying Differentially Methylated Regions between unique samples using array based methylation profiles
Source: BMC Bioinformatics. 2014 May 15;15:141. doi: 10.1186/1471-2105-15-141 (PMC4046028; doi:10.1186/1471-2105-15-141)
Supplement: Additional file 2 — DMRforPairs output for the comparison of A431-MCF7 and NA17018-NA17105. Please start from the HTML files in each folder. Available via the BMC Bioinformatics website. [file 1471-2105-15-141-S2.zip › 1394847754114233_MOESM2_ESM/A431_MCF7/figures/19883.pdf]

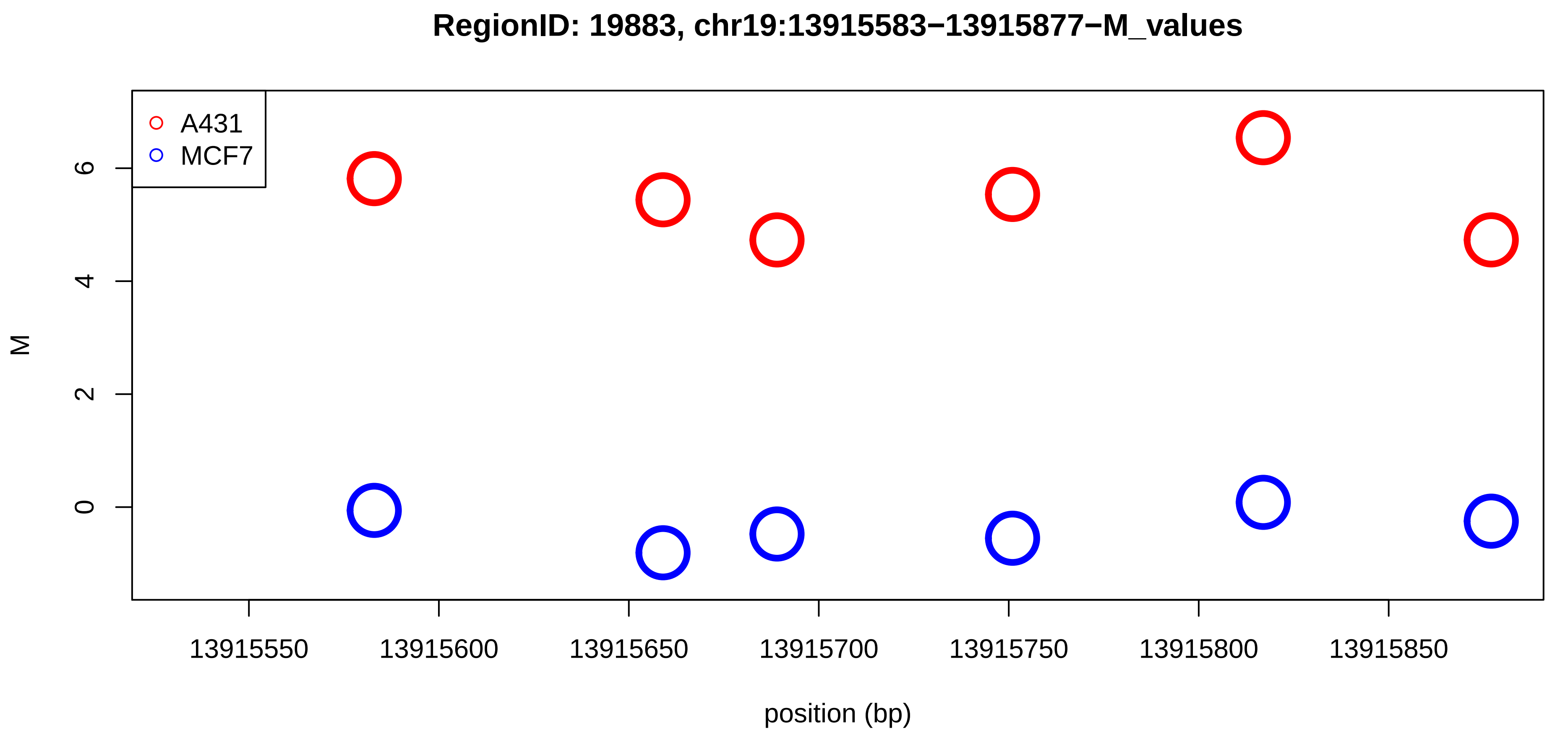

RegionID: 19883, chr19:13915583−13915877−M_values
A431
MCF7
M
6
4
2
0
13915550
13915600
13915650
13915700
13915750
13915800
13915850
position (bp)

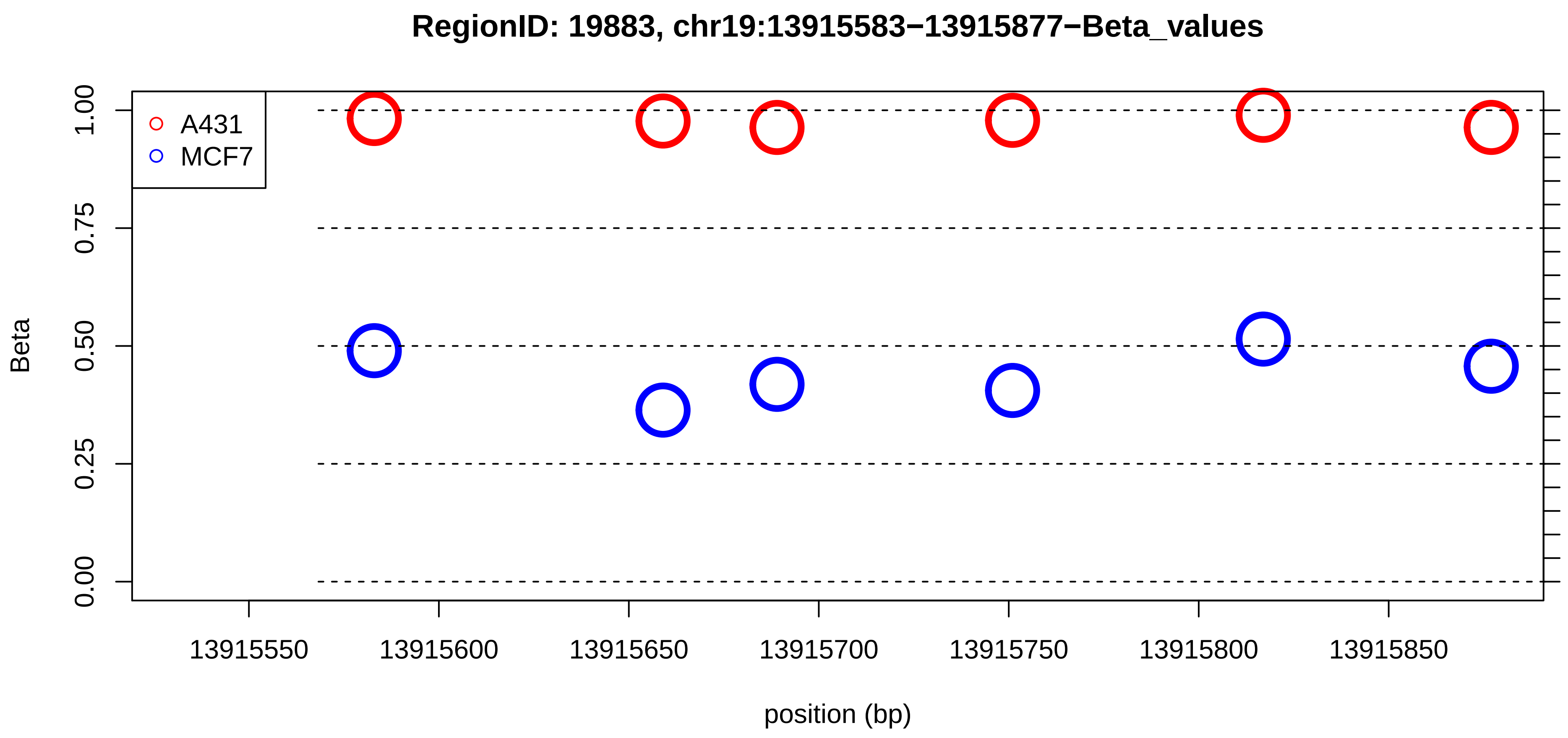

RegionID: 19883, chr19:13915583−13915877−Beta_values
A431
MCF7
Beta
1.00
0.75
0.50
0.25
0.00
13915550
13915600
13915650
13915700
13915750
13915800
13915850
position (bp)

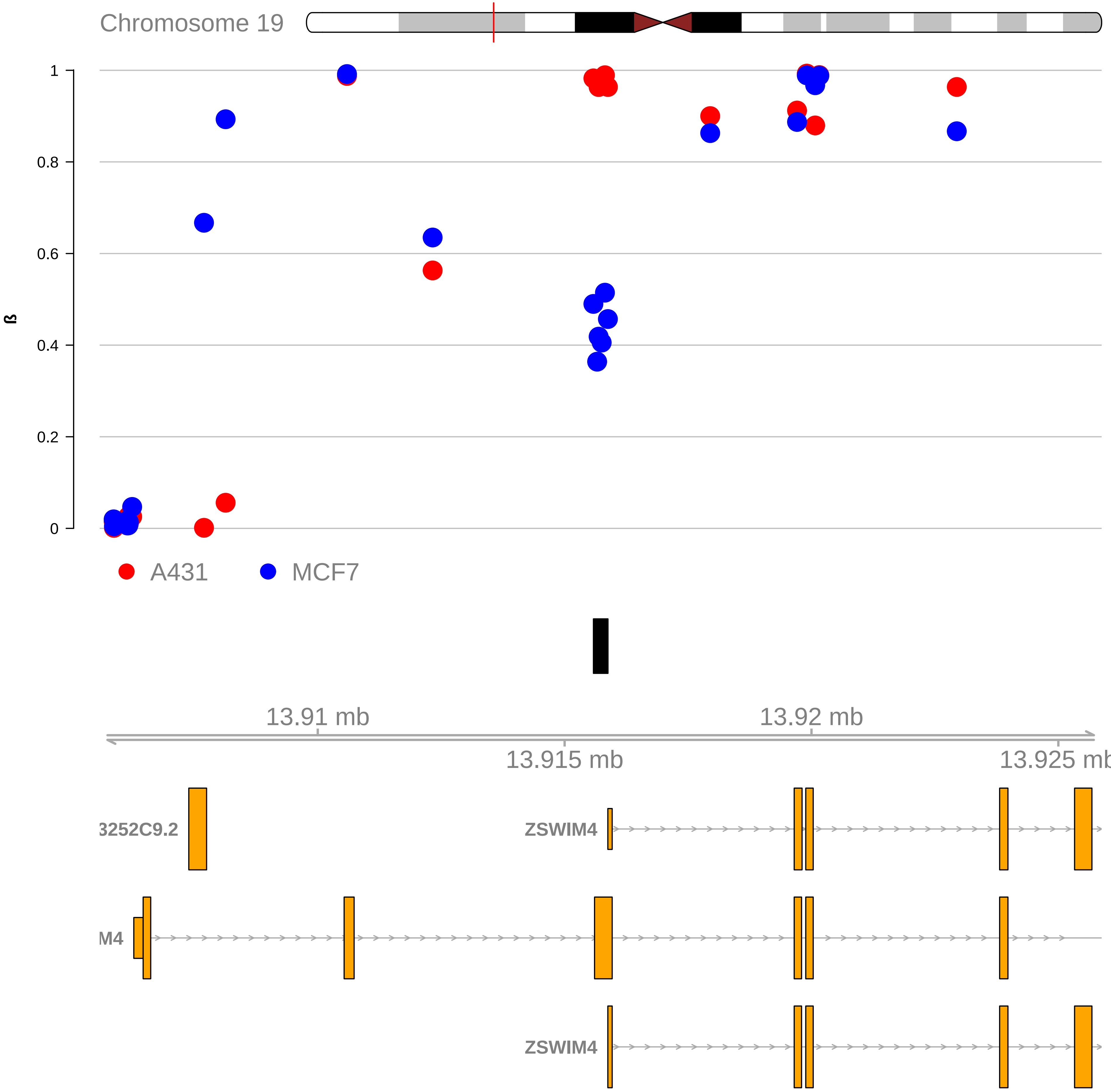
Chromosome 19
1
0.8
0.6
0.4
0.2
0
ß
A431
MCF7
13.91 mb
13.92 mb
13.915 mb
13.925 mb
3252C9.2
ZSWIM4
M4
ZSWIM4
